# Late-onset toxic anterior segment syndrome after ICL implantation: two case reports

**DOI:** 10.1186/s12886-022-02713-3

**Published:** 2023-02-11

**Authors:** Li Li, Qizhi Zhou

**Affiliations:** Refractive Surgery Center, Chongqing Eye and Vision Care Hospital, Yuzhong District, NO.77, the second Changjiang Road, Chongqing, China

**Keywords:** Toxic anterior segment syndrome, V4c Implantable Collamer Lens

## Abstract

**Background:**

Toxic anterior segment syndrome (TASS) is a non-infectious inflammation that can occur after any anterior segment procedure. This case report presents two relatively rare late-onset TASS cases after V4c implantable collamer lens (ICL) operation.

**Case presentation:**

One 25-year-old woman and one 31-year-old woman suddenly had vision loss in monocular for 1 week after biocular V4c ICL operations and with no subjective complaints. They both presented fibrin formation in the anterior chamber such as keratic precipitates and white pus on the surface of the ICL. Fundus examination was normal. After 4 to 5 weeks of topical and oral steroid treatment, visual acuity and fibrin formation in the anterior chamber improved during the follow-up.

**Conclusions:**

TASS should be suspected in any patient during the late period following ICL surgery; Once TASS is diagnosed, adequate treatment with intensive steroid therapy can be implemented.

## Background

In recent years, implantable collamer lenses (ICL) have become increasingly common for the treatment of ametropia, and more than one million ICLs have been implanted worldwide. Previous studies have shown that V4c ICL with a central hole performed well in terms of safety, efficacy, predictability, and stability over a long-term follow-up period [1, 2]. There are, however, still some complications that need to be addressed, such as toxic anterior segment syndrome (TASS), a non-infectious inflammation that can occur after any anterior segment operation [3]. In this case report, we presented two relatively rare cases of late-onset TASS, which occurred after V4c ICL implantation.

## Case presentation

### Case 1

A 25-year-old woman presented to our department for refractive surgery. Before surgery, the patient’s uncorrected distance visual acuity (UDVA) was 1/25 in the right eye (OD), and 1/50 in the left eye (OS) which improved to 20/25 in both eyes (OU) with a refraction of −12.75 diopter sphere (DS)/−2.25 diopter cylinder (DC)×175° OD and −14.25DS/−1.00 DC×180° OS. Anterior segment examination results were normal, and the patient’s intraocular pressure (IOP) was 16.9 mmHg OD and 17.2 mmHg OS. The central corneal thickness was 582 µm OD and 583 µm OS, and the microscopy endothelial cell count was 2,527 cells/mm^2^ OD and 2,555 cells/mm^2^ OS. The corneal topography (Pentacam HR, Oculus) results were normal in both eyes. The anterior chamber depth was 2.96 mm OD and 3.04 mm OS, and the white-to-white diameters were 11.8 mm OD and 11.7 mm OS. Fundus examination revealed changes of myopia in both eyes, with no peripheral treatable lesions. On August 10, 2021, the patient underwent a V4c ICL implantation (−14.5 DS; overall diameter, 12.6) OD under topical anesthesia (0.4% oxybuprocaine hydrochloride eye drops), followed by the implantation of a V4c ICL (-15.0 DS/ +1.50 DC at 91°; overall, 12.6) OS two days later. Intraoperatively, 1% viscocohesive sodium hyaluronate and 0.5% tropicamide phenylephrine eye drops were used, while 0.5% levofloxacin sodium hyaluronate, and 0.2% brimonidine tartrate eye drops were routinely used for the first week after surgery.

**Fig. 1:**
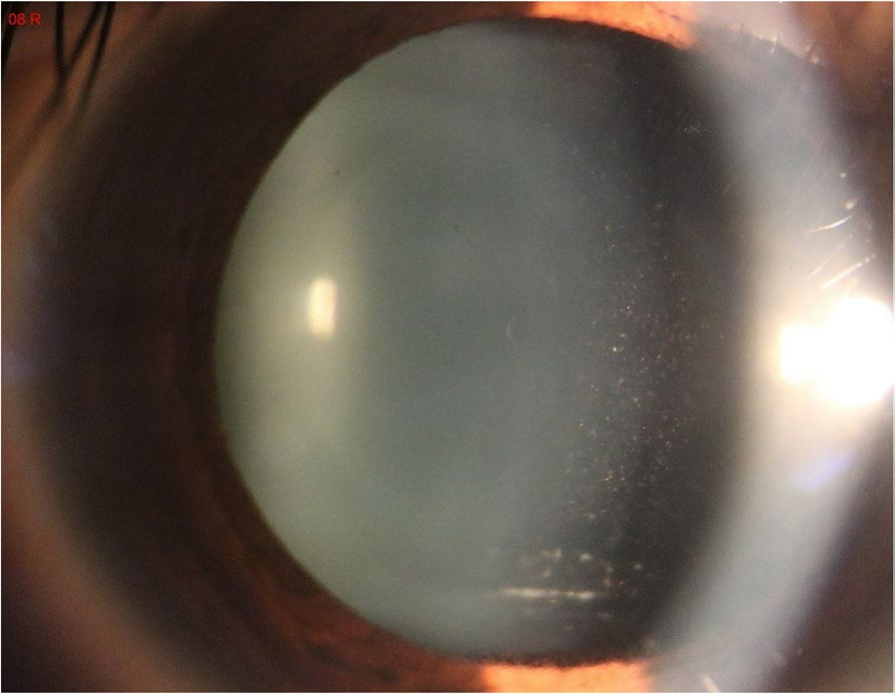
Slitlamp photograph ( x16 magnification) of the right eye of Case 1

On postoperative day 1, the patient’s OU UDVA was 20/20, and the anterior segment of both eyes was normal. At 1 week postoperative, her UDVA was 20/32 in the right eye and 20/20 in the left, and there were no physical signs. In her right eye, keratic precipitates (KP) were observed on the posterior surface of the cornea, while cells and flares were observed in the anterior chamber. Additionally, numerous pigments and white pus were observed on the surface of the ICL (Fig. 1). The IOP was 13 mmHg OD and 14 mmHg OS, and fundus results were normal.

The patient was treated with prednisolone (0.5 mg/kg), 1% topical prednisolone hourly, 0.25% topical amikacin every 6h, and 5% topical levofloxacin every 6h. On one day of treatment, the number of cells in the anterior chamber was significantly decreased, and three days after treatment, the patient’s UDVA was 0.8 and her IOP was 19.7 mmHg. At one week of treatment, the anterior chamber was clear and the white pus on the surface of ICL was significantly decreased. Topical antibiotics and oral steroids were administered for the first two weeks after surgery, and topical prednisolone was tapered slowly over the next four weeks.

**Fig. 2:**
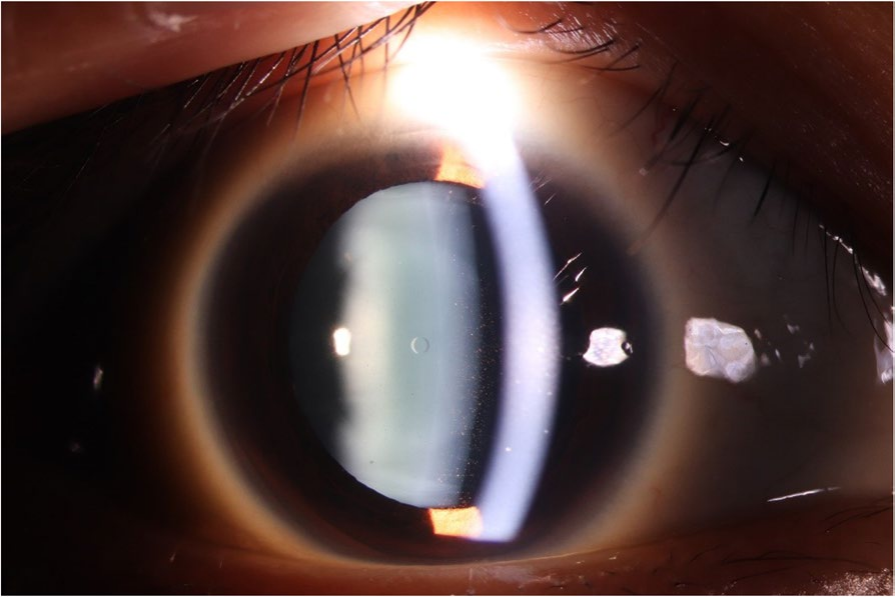
Slitlamp photograph ( x10 magnification) of the right eye of Case 1

At 5 weeks post-op, the OD UDVA was 20/25, which was comparable to the patient’s preoperative corrected distance visual acuity. The cornea of the right eye was clear, the IOP was 14.0 mmHg. The central corneal thickness was 580 µm, and the endothelial cell count was 2,530 cells/mm^2^. Some pigments were, however, observed on the surface of the ICL (Fig. 2).

### Case 2

A 31-year-old woman presented to our department for refractive surgery. The UDVA was 20/250 OD and 20/320 OS, which improved to 20/25 OU, with a refractive correction of −8.50 DS OD and −6.25DS/−3.25 DC×20° OS. Anterior segment examination results were normal, and the IOP was 13.0 mmHg OU. The central corneal thickness was 493 µm OD and 492 µm OS, and the endothelial cell count was 2,818 cells/mm^2^ OD and 2,796 cells/mm^2^ OS. The OU corneal topography was normal, and the anterior chamber depth was 2.87 mm OD and 2.82 mm OS. The white-to-white diameter was 11.9 mm OD and 12.0 mm OS, and the fundus examination did not reveal any treatable lesions. The patient underwent implantation of V4c ICL (−9.0 DS; overall diameter, 13.2) in the right eye on Aug 14, 2021, and then in the left eye (-10.0 DS/ +3.0 DC at 102°, overall, 12.6) two days later.

**Fig. 3:**
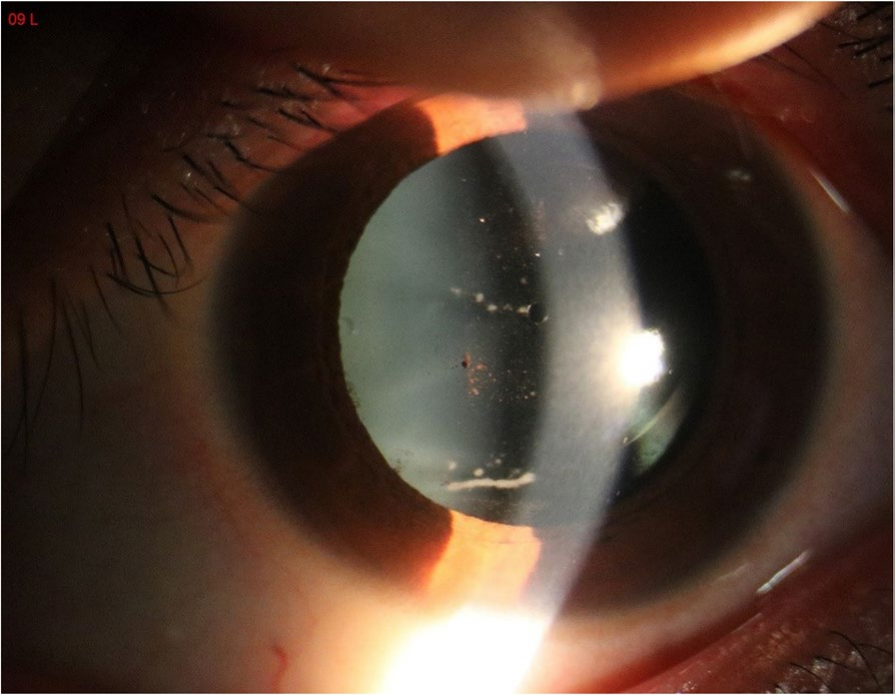
Slitlamp photograph ( x10 magnification) of the left eye of Case 2

On 1 day postoperatively, the OU UDVA was 20/20, and the anterior segment was normal in both eyes. One week after surgery, the UDVA of the left eye was 20/25, while that of the right eye was 20/20, and the patient reported no complaints. There was asmall KP on the posterior surface of the cornea and amounts of cells and flares in the anterior chamber. A large number of pigments and white pus were observed on the surface of the ICL lens (Fig. 3). The patient’s IOP was 10.3 mmHg OD and 10.0 mmHg OS, and the fundus examination was normal.

The patient was treated with oral prednisolone (0.5 mg/kg), 1% topical prednisolone hourly, 0.25% topical amikacin 6h, and 5% topical levofloxacin 6h. After treatment, the number of cells in the anterior chamber decreased within the next 5 days. At 1 week of treatment, the anterior chamber was clear, and the white pus on the surface of the lens was also significantly decreased. The UDVA at day 7 after treatment was 20/20 with a relative improvement in signs. The topical antibiotics and oral steroids were continued for the first 2 weeks. The topical prednisolone was then tapered slowly over the next 4 weeks.

**Fig. 4:**
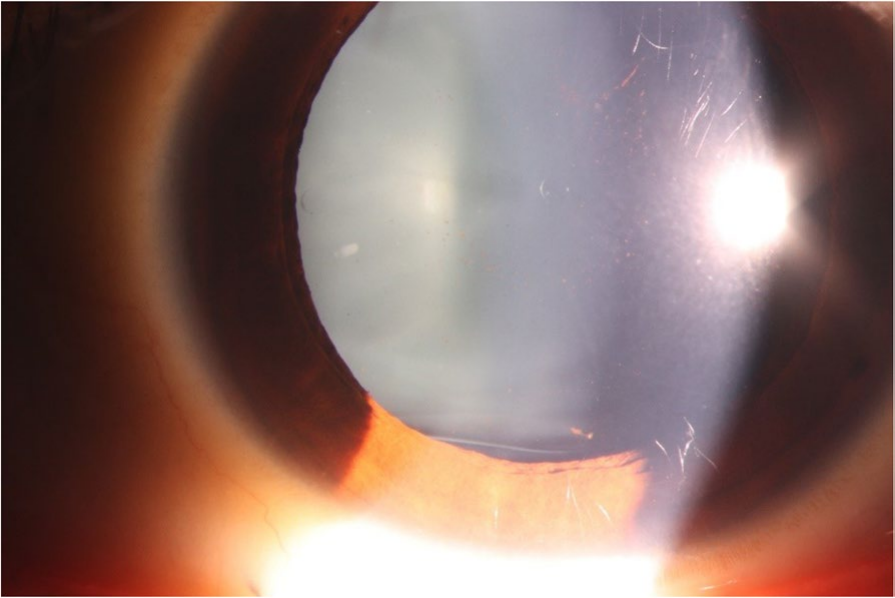
Slitlamp photograph ( x16 magnification) of the left eye of Case 2

After 4 weeks post-op, the OU UDVA was 20/20, the IOP was 12.3 mmHg OD, and 12.1 mmHg OS. The central corneal thickness was 491 µm of the left eye, and the endothelial cell count was 2,789 cells/mm^2^. Some pigments were observed on the surface of the ICL lens (Fig. 4).

## Discussion and conclusion

TASS is a non-infectious inflammation that can occur after any anterior segment surgery. A previous study described some typical symptoms of TASS, including corneal edema, fibrin formation, and glaucoma, while also indicating that TASS typically occurs after the first 12-48 hours post-op [4]. Some late-onset cases of TASS were found to have occurred after an average of 38.44 days post-op [5]. In the present study, we have described two late-onset cases of TASS within 1 week after ICL implantation. To the best of our knowledge, this is the first case report involving incidents of late-onset TASS after ICL implantation.

A previous retrospective study described 60 eyes diagnosed with TASS resulting from 26,408 cataract surgeries (0.22%) [6], and based on data from the TASS task force, which was formed by the American Society of Cataract and Refractive Surgery (ASCRS), there were 1,454 cases of TASS resulting from 66,000 procedures performed between June 2007 and March 2012. The incidence of TASS after ICL implantation has not been previously reported, although, based on a prospective study by Sherif et al., 1 eye out of 54 (1.85%) was found to have TASS after V4c ICL implantation [7]. According to data from our refractive center, 827 eyes were implanted with V4c ICL from January 2021 to September 2021, of which 2 were diagnosed with TASS (0.24%), a lower incidence than previously reported.

Some previous studies about TASS after V4c ICL surgery [8, 9] indicated that TASS often occurred 1d postoperatively, and corneal edema and anterior chamber reactions were the most common sign in these case reports. In the cases we have described herein, TASS occurred later, with patients experiencing blurry vision and fibrin formation on 1-week post-op. There was, however, less corneal edema in the cases we have described, and the pathology may be different from that usually found with TASS. Other previous studies indicated that the proportion of corneal edema in late-onset TASS was only 15.6%~19.1% [5, 10].

Based on a previous study, the risks for TASS included intraocular lens and solution, ocular viscoelastic materials, balanced salt solution, antibiotics, anesthetics, intraocular dye, ointments, etc. [11]. Outbreaks in some surgery centers may be related to specific risks, although most cases have no clear risks. In the two TASS cases we have described herein, the surgeries were performed on separate days. We reviewed seven other patients who underwent ICL surgeries on the same day and found no other reported incidents of TASS. During the operation, we used naphazoline hydrochloride, chlorphenamine maleate and vitamin B12 eye drop to contract the blood vessels after conjunctival sac flushing using Shike® compound electrolyte intraocular irrigating solution. Additionally, the naphazoline hydrochloride used in our center had less NaOH, although based on a previous study, NaOH may be a risk factor for TASS [12]. We didn’t test the PH value or other special components of aqueous humor, which may help determine the exact risk factors for TASS.

The present study did have some limitations. First, we couldn’t identify the exact risk factors that caused TASS. The only correlation we could find was that the usage of naphazoline hydrochloride may change the PH of the ocular. Second, post-op endophthalmitis was found to be a close and differential diagnosis of TASS; however, the site of the inflammatory reaction is generally in the anterior segment in TASS, which is less associated with eye pain symptoms, sensitive to hormone therapy, and diagnosed based on negative bacterial culture results. In the present study, we did not test for any bacteria. Moreover, in the two cases presented herein, as they were monocular and involved treatment with intense topical corticosteroid over a period of months, which resulted in the resolution of the anterior chamber flare and the white pus on the surface of the ICLs, these cases were diagnosed as TASS.

In conclusion, TASS should be suspected in any patient, whether in the early or late period following ICL surgery, especially when there is no risk of postoperative inflammation. Late-onset TASS may result in less corneal edema, and to prevent TASS, the focus should be on adequate surgical procedures involving the use of intraocular medications, solutions, and eye drops. Once TASS is diagnosed, adequate treatment with intensive steroid therapy can be implemented.

## Data Availability

The datasets used and/or analyzed during the current study available from the corresponding author on reasonable request.

## Abbreviations

ICL: Implantable collamer lens
TASS: Toxic anterior segment syndrome
